# Carbohydrates protect protein against abiotic fragmentation by soil minerals

**DOI:** 10.1038/s41598-017-19119-7

**Published:** 2018-01-16

**Authors:** Patrick N. Reardon, Eric D. Walter, Carrie L. Marean-Reardon, Chad W. Lawrence, Markus Kleber, Nancy M. Washton

**Affiliations:** 1 Environmental Molecular Science Laboratory, Pacific Northwest National Laboratory, Richland, WA 99354 USA; 2 School of the Environment, Washington State University Tri-Cities, Richland, WA 99352 USA; 3 Department of Crop and Soil Science, Oregon State University, Corvallis, OR 97331 USA; 4 OSU NMR Facility, Oregon State University, Corvallis, OR 97331 USA

## Abstract

The degradation and turnover of soil organic matter is an important part of global carbon cycling and of particular importance with respect to attempts to predict the response of ecosystems to global climate change. Thus, it is important to mechanistically understand the processes by which organic matter can be degraded in the soil environment, including contact with reactive or catalytic mineral surfaces. We have characterized the outcome of the interaction of two minerals, birnessite and kaolinite, with two disaccharides, cellobiose and trehalose. These results show that birnessite reacts with and degrades the carbohydrates, while kaolinite does not. The reaction of disaccharides with birnessite produces Mn(II), indicating that degradation of the disaccharides is the result of their oxidation by birnessite. Furthermore, we find that both sugars can inhibit the degradation of a model protein by birnessite, demonstrating that the presence of one organic constituent can impact abiotic degradation of another. Therefore, both the reactivity of the mineral matrix and the presence of certain organic constituents influence the outcomes of abiotic degradation. These results suggest the possibility that microorganisms may be able to control the functionality of exoenzymes through the concomitant excretion of protective organic substances, such as those found in biofilms.

## Introduction

Soil organic matter (SOM) represents the largest carbon reservoir in the terrestrial biosphere^1,2^ and its representation in Earth System Models (ESM) continues to pose a significant challenge for the development of robust carbon cycle and climate projections.^3^ Changes in soil organic carbon were found to be related to the strength of simulated carbon-climate feedbacks^4^, emphasizing the need for an improved understanding of the mechanisms involved in retarding or accelerating the turnover of soil carbon. The enzymatic disassembly of large organic debris is probably the most critical of all the soil carbon cycling processes that need to be better understood^5^. It is generally assumed that the high- molecular-weight (HMW) fraction of organic matter is initially hydrolyzed by extracellular enzymes to sizes <600 Da^6^ for transport into the cell^7^. While exceptions to this rule seem possible^8^, extracellular enzymatic disassembly of large organic debris should be considered a necessity to enable passage of the microbial cell wall for compounds larger than 600–1000 Da^9^. Together with recent evidence pointing at a prominent role of habitat properties for microbial decomposition^10–12^, this insight creates the need to better understand the role of the soil matrix as a constraint for extracellular enzymatic activity.

Minerals comprise the bulk of most soils, making their interactions with organic matter important to understanding the cycling of organic matter in soil. Phyllosilicate clays and other solid surfaces have been shown to bind proteins and other biomolecules^13^. In some cases, enzymes can remain active in the adsorbed state^13–16^. Increased resistance to thermal denaturation has also been observed for proteins bound to some solid surfaces^13^. More generally, natural organic matter has been shown to adsorb to minerals, with some minerals exhibiting preferential binding for certain constituents of natural organic matter^17^.

Mineral constituents of soils can have significantly different surface chemistries and reactivities. For example, the metal oxide mineral birnessite, a strongly oxidizing manganese oxide, has previously been shown to oxidize small organic compounds^18–20^. It has also been shown to adsorb and oxidize natural organic matter, although the specific compounds in the organic matter that are oxidized were not identified^17^. More recent analysis of fungal mediated decomposition of forest litter has shown that reactive Mn(III) may contribute to the decomposition of litter constituents such as lignin, accompanied by the subsequent formation of Mn(III,IV) oxides^21^. Finally, recent studies have shown that birnessite can fragment proteins, in environmentally relevant conditions, resulting in the production of soluble peptides^22,23^. These observations establish the fundamental ability of manganese oxides to participate in a wide range of chemical processes that are pertinent to the soil environment.

Carbohydrates are significant components of soil organic matter as well as dissolved organic matter and are a major pool of carbon available for interaction with minerals^24^. The goal of this study is to determine the extent to which carbohydrates react with two minerals, kaolinite and birnessite, and how this reaction could influence the fragmentation of protein by birnessite. To achieve this goal, we reacted two disaccharides, cellobiose and trehalose, with kaolinite and birnessite. Trehalose is a non-reducing sugar that is found throughout the tree of life and can accumulate in some organisms to ~7% of dry weight^25^. Trehalose has been identified in abundance in environmental samples^26^, including biofilms^27^. Cellobiose is produced during the depolymerization of cellulose, a major source of input carbon to soil, and is metabolized by soil biota for energy^28^. Cellobiose is a reducing sugar with a free hemi-acetal that impacts its reactivity. In addition, we examined the ability of these carbohydrates to inhibit the reaction between a model protein, GB1, and birnessite. The minerals were chosen to represent two different categories of reactivity towards organic compounds. The pedogenic oxide birnessite has the well documented ability to oxidize organic molecules^18,19,29,30^ and to catalyze the fragmentation of protein into small peptides^22,23^. The phyllosilicate kaolinite is typically well crystalline, rendering basal planes largely hydrophobic, while platelet edges tend to be decorated with hydroxyls coordinated to Al cations. As a result, kaolinite tends to act as a sorbent for organic compounds, especially for polyelectrolytes possessing significant hydrophobic regions^31^. Both manganese oxide (birnessite) and kaolinite can be considered ubiquitous in most soils around the globe^32^. The products of these reactions were analyzed with nuclear magnetic resonance (NMR) spectroscopy to determine the extent of carbohydrate reaction with birnessite and whether the presence of carbohydrate affected the interaction between protein and birnessite. Electron paramagnetic resonance (EPR) spectroscopy was used to determine the amount of Mn(II) released from the birnessite during the reaction with the carbohydrates.

## Materials and Methods

Trehalose and cellobiose were added to 20 mg of birnessite or kaolinite in 1 mL of milliQ H_2_O. The desired pH of each sample was adjusted using HCl and NaOH. Samples were incubated at 25 °C for 24 hours with constant agitation. Following the incubation, minerals were removed by centrifugation at 21,000xg for 5 minutes. Supernatants were removed and centrifuged at 21,000 × g for 5 minutes to remove residual solid minerals. 540 µL were carefully removed from the samples without disturbing the residual mineral pellet. D_2_O and DSS were added to a final concentration of 10% v/v and 0.5 mM respectively and either stored at −80 °C or used immediately for NMR analysis. The concentration of formate produced from the reactions with birnessite was estimated from a total of three repeated experiments under each condition. The birnessite and kaolinite used in this study were the same as those used in our previous study and were characterized as previously described^23^.

^15^N stable isotope labeled protein G, B1 domain (GB1) was expressed and purified from BL21(DE3) *E. coli* cells as previously described^23,33^. 0.4 mg of GB1 was combined with 20 mg birnessite and increasing concentrations of trehalose, up to 100 mM. Samples were incubated at 25 °C for 24 hours with constant agitation. Minerals were removed from the sample using the same method as the samples without protein. D_2_O and DSS were added to a final concentration of 10% v/v and 0.5 mM respectively. Samples were immediately analyzed with NMR spectroscopy.

NMR data were collected using either a 600 MHz or 800 MHz Agilent VNMRS spectrometer equipped with cryogenic triple resonance probes. The ^1^H experiments were collected using the recommended parameters for the Chenomx software to enable accurate concentration estimates. A 1D NOESY experiment was used with a spectral window of 12 ppm, 4 s acquisition time and 1 s recycle delay. 2D ^15^N-HSQC experiments were collected with 1024 complex points in the direct dimension and 128 complex points in the indirect dimension. Sweep widths were 16 ppm in the direct dimension and 35.86 ppm in the indirect dimension. All NMR data were collected at 25 °C. Data were processed, apodized and phased in nmrPipe^34^ and analyzed using Chenomx (1D proton) or NMRViewJ^35^ (2D HSQC). Formate concentrations determined using Chenomx are summarized in Table 1.

**Table 1:** Chenomx based estimates of formate concentrations produced by reaction of 1 mM carbohydrate with birnessite

Carbohydrate	pH	Formate (mM)	Standard deviation
Cellobiose	5	3.8	0.2
Cellobiose	7	4.6	0.4
Trehalose	5	1.9	0.2
Trehalose	7	2.1	0.1

### EPR spectroscopy

Electron paramagnetic resonance (EPR) spectra were acquired with a Bruker Elexsys 580 spectrometer fitted with an SHQE resonator and a Bruker continuous flow nitrogen VT insert. Room temperature aqueous samples were contained in 1.0 mm OD × 0.8 mm ID quartz tubes (Vitrocom) sealed with Critoseal capillary tube sealant (Leica Microsystems). These were placed in conventional 4 mm OD × 3 mm ID quartz EPR tubes (Wilmad). The microwave frequency was ~9.86 GHz (~9.33 GHz without the VT insert), and microwave power was 20 mW. The field was swept at 1000 Gauss (G) in 84 s and modulated at a frequency of 100 kHz with 5 G amplitude. A time constant of 82 ms was employed, and typically 20 scans were averaged. Quantitation of Mn(II) reprised the method of Reardon *et al*.^23^.

### Data availability

The data generated in this study are available from the corresponding author on reasonable request.

## Results and Discussion

**Figure 1:**
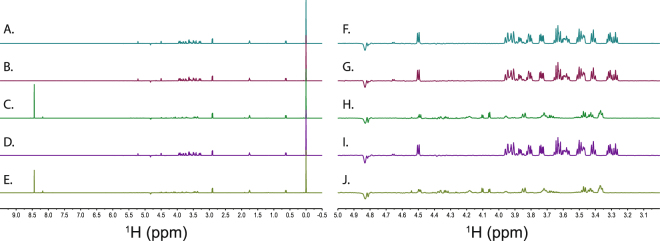
^1^H NMR spectra of cellobiose reaction products with birnessite and kaolinite at pH 5 and pH 7. Panels A through E show the full spectrum at each reaction condition. Panels F through J focus on the carbohydrate region of the spectrum, 3.0 ppm to 5.0 ppm. (**A** and **F**) are 1 mM cellobiose without added mineral. (**B** and **G**) are 1 mM cellobiose in the presence of kaolinite at pH 7. (**C** and **H**) are 1 mM cellobiose in the presence of birnessite at pH 7. (**D** and **I**) are 1 mM cellobiose in the presence of kaolinite at pH 5. (**E** and **J**) are 1 mM cellobiose in the presence of birnessite at pH 5.

**Figure 2:**
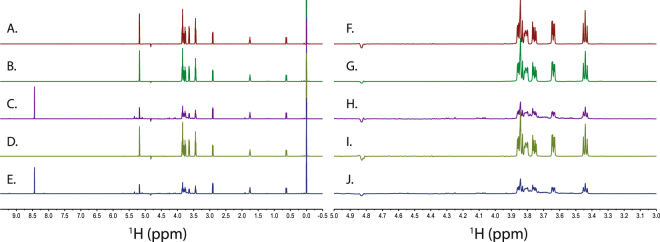
^1^H NMR spectra of trehalose reaction products with birnessite and kaolinite at pH 5 and pH 7. Panels A through E show the full spectrum at each reaction condition. Panels F through J focus on the carbohydrate region of the spectrum, 3.0 ppm to 5.0 ppm. (**A** and **F**) are 1 mM trehalose without added mineral. (**B** and **G**) are 1 mM trehalose in the presence of kaolinite at pH 7. (**C** and **H**) are 1 mM trehalose in the presence of birnessite at pH 7. (**D** and **I**) are 1 mM trehalose in the presence of kaolinite at pH 5. (**E** and **J**) are 1 mM trehalose in the presence of birnessite at pH 5.

Solution state ^1^H NMR spectroscopy revealed that both trehalose and cellobiose participated in chemical reactions with birnessite. This is demonstrated by the appearance of new proton resonances in the NMR spectrum that do not correspond to the starting material, as shown in Figs 1 and 2. These resonances were not observed in minerals without the carbohydrate, nor were they observed in the carbohydrates without mineral when allowed to incubate under the same conditions. Thus, birnessite is capable of reacting with and degrading carbohydrates under pH and temperature conditions similar to those found in the soil environment. In reactions with birnessite and each sugar, we observed a strong peak at a proton chemical shift of ~8.2 ppm. The chemical shift of this peak is consistent with the production of formate. Identification of the peak as formate was confirmed by spiking the sample with 1 mM formic acid and observing that the candidate peak increased in intensity and shifted due to the change in pH.

In contrast to birnessite, the NMR spectra of trehalose and cellobiose following a 24 hour reaction with kaolinite were essentially identical to the unreacted samples as shown in Figs 1 and 2. This indicates that kaolinite does not react with or catalyze the degradation of these disaccharides under the conditions used for our experiments. These spectra indicated that the carbohydrate concentrations were essentially identical to the starting concentrations, indicating minimal binding of the carbohydrates by kaolinite. This is consistent with previous results which show that adsorption of cellobiose and glucose by kaolinite is not significant^15^. Our results show that this is also true for the non-reducing sugar trehalose, suggesting that small carbohydrates are unlikely to benefit from kaolinite mediated protection from degradation. Results at pH 7 were similar to those observed at pH 5.

**Figure 3:**
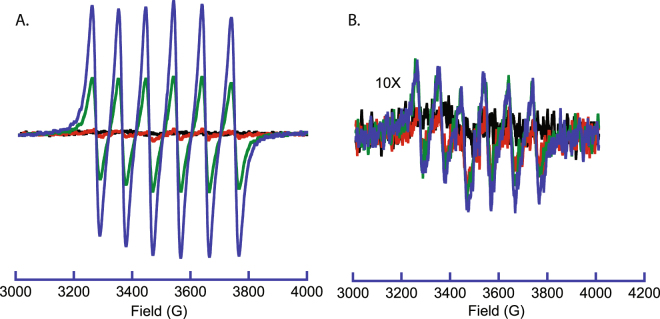
EPR analysis of Mn(II) production during reaction of birnessite with carbohydrates. (**A**) Cellobiose. (**B**) Trehalose (Y-axis scaled 10X). The presence of Mn^2+^ is indicated by the observation of 6 EPR lines with concentration-dependent signal intensities. Concentrations of carbohydrate are 0 mM (Black), 10 mM (Red), 50 mM (Green), 100 mM (Blue).

Production of Mn(II) in the birnessite reactions was monitored by EPR spectroscopy. Oxidation of small organic compounds by birnessite has been observed to produce Mn(II) in solution^18^. Incubation of birnessite with either sugar produced increasing concentrations of Mn(II) with increasing concentrations of carbohydrate as shown in Fig. 3. These data show that the reaction of birnessite with sugars involves reduction of Mn(III, IV) to Mn(II) and its subsequent release into solution, consistent with previous results for small organic compounds. The degradation of cellobiose produced more Mn(II) when compared to like concentrations of trehalose, as indicated by the overall higher intensities of Mn(II) EPR spectrum in the cellobiose treated samples.

**Figure 4:**
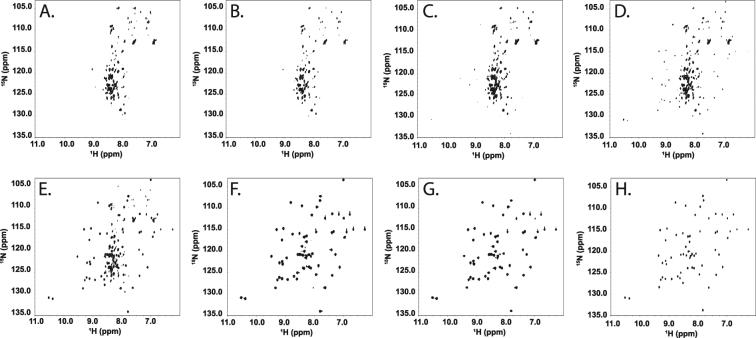
^15^N HSQC NMR spectra of birnessite-GB1-cellobiose reactions. (**A**) through (**G**). Increasing concentrations of cellobiose (0 mM, 0.5 mM, 1 mM, 5 mM, 10 mM, 50 mM and 100 mM respectively) were added to GB1 protein (64.5 μM) and birnessite at ph 5 and allowed to react for 24 hours. (H) GB1 spectrum without mineral or cellobiose.

**Figure 5:**
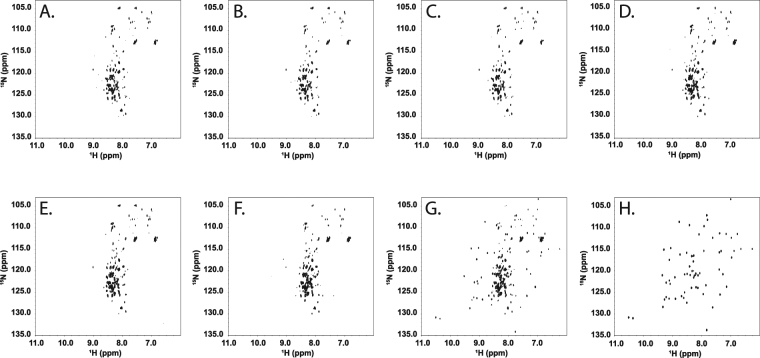
^15^N HSQC NMR spectra of birnessite-GB1-trehalose reactions. (**A**) through (**G**) Increasing concentrations of trehalose (0 mM, 0.5 mM, 1 mM, 5 mM, 10 mM, 50 mM and 100 mM respectively) were added to GB1 protein (64.5 μM) and birnessite at pH 5 and allowed to react for 24 hours. (H) GB1 spectrum without mineral or trehalose.

To determine the impact that sugars have on birnessite mediated degradation of proteins, we have examined the reaction of the model protein GB1 with birnessite in the presence of trehalose and cellobiose. The ^1^H and ^15^N chemical shifts obtained from the 2D ^15^N-HSQC NMR experiment are sensitive to the three dimensional structure of a protein and are commonly used to assess the structural integrity of proteins^36–38^. This technique has previously been shown to be a valuable tool for analyzing protein degradation by birnessite, where birnessite catalyzes the hydrolytic degradation of GB1^23^. 2D ^15^N-HSQC NMR spectra of the birnessite, carbohydrate and GB1 reaction supernatants revealed that both trehalose and cellobiose inhibit the degradation of protein by birnessite in a dose dependent manner. Intact and folded protein was unambiguously identified in the supernatant of reactions with ~5 mM cellobiose as demonstrated by the appearance of NMR resonances corresponding to folded GB1, shown in Fig. 4. For trehalose, intact and folded protein was only observed in the reaction with 100 mM trehalose, which is shown in Fig. 5. Thus, cellobiose is both a better inhibitor of birnessite catalyzed protein degradation and a better reactant with birnessite when compared to trehalose.

**Figure 6:**
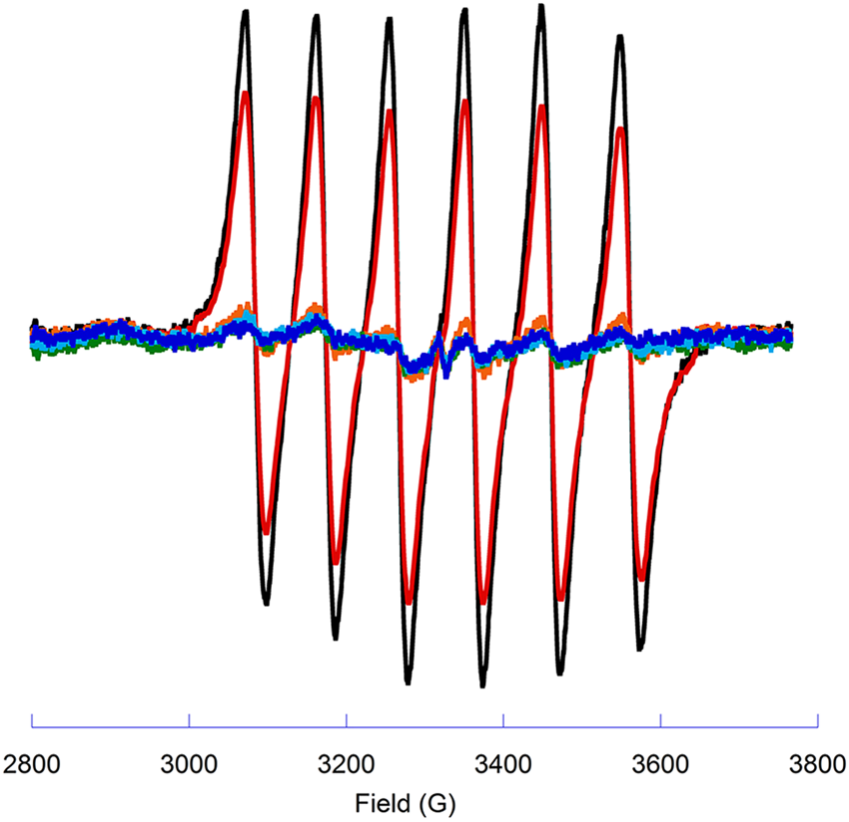
EPR analysis of Mn(II) production during reaction of birnessite with cellobiose in the presence of increasing concentrations of GB1 protein. The presence of Mn^2+^ is indicated by the observation of 6 EPR lines with concentration-dependent signal intensities. Concentrations of GB1 were 0 mg/ml (black), 0.05 mg/ml (red), 0.1 mg/ml (orange), 0.2 mg/ml (green), 0.4 mg/ml (light blue) and 0.8 mg/ml (dark blue). The concentration of cellobiose was 10 mM (3.4 mg/ml).

**Figure 7:**
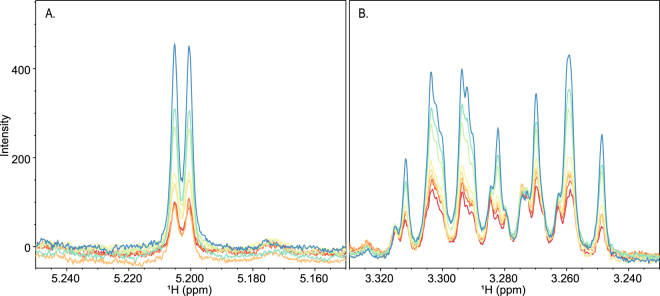
Inhibition of birnessite mediated cellobiose degradation by GB1. Two regions of the 1D ^1^H NMR spectrum, 5.15 ppm to 5.25 ppm (**A**) and 3.22 ppm to 3.34 ppm (**B**), which contain isolated resonances corresponding to cellobiose, are shown. The colors indicate the concentration of GB1 used in the experiment; 0 mg/ml GB1 (dark red), 0.01 mg/ml GB1 (light red), 0.025 mg/ml GB1 (orange), 0.05 mg/ml GB1 (light orange), 0.1 mg/ml GB1 (yellow), 0.2 mg/ml GB1 (yellow-green), 0.4 mg/ml GB1 (aqua), 0.8 mg/ml GB1 (blue). Initial cellobiose concentrations were 10 mM (3.4 mg/ml).

We further examined the impact of GB1 on the reduction of birnessite by cellobiose. We have previously shown that GB1 does not significantly promote the reduction of birnessite to Mn(II). Using EPR spectroscopy, we found that GB1 inhibited the production of Mn(II) when 10 mM cellobiose (10 mM) reacted with birnessite, as shown in Fig. 6. Inhibition of Mn(II) production was accompanied by a reduction in the amount of cellobiose consumed during the reaction, as determined by NMR spectroscopy and shown in Fig. 7. Taken together, these data suggest that GB1 and cellobiose may interact with similar reactive sites on the birnessite surface. The molar concentration of GB1 is significantly less than cellobiose or trehalose in all of our reaction conditions where inhibition was observed. This suggests that GB1 likely has a much stronger binding affinity for birnessite than cellobiose or trehalose, under the reaction conditions tested.

Trehalose and cellobiose are glucose dimers that differ in their glycosidic linkages. Cellobiose consists of two glucose molecules linked by a β(1–4) glycosidic linkage. Cellobiose is a reducing sugar, having a free hemiacetal that can form a reactive aldehyde. In contrast, trehalose consists of two glucose molecules linked by an α(1–1) glycosidic linkage, which converts both hemiacetals in the glucose monomers to acetals, making it a non-reducing sugar. In our experiments, cellobiose was consistently a better reactant with birnessite, and less cellobiose (5 mM) was required to inhibit the birnessite mediated protein degradation when compared to trehalose (100 mM). The reactive hemiacetal likely makes cellobiose more susceptible to birnessite mediated oxidation, resulting in increased reaction with birnessite compared to trehalose^39^. When compared to trehalose, the increased reactivity of cellobiose with birnessite is likely to contribute to its improved ability to prevent protein degradation by birnessite. Cellobiose could be reacting with catalytic surface sites on the birnessite, resulting in conversion of those sites to catalytically inactive sites and inhibition of protein degradation. Recent molecular dynamics simulations have suggested that protein degradation by birnessite may involve out-of-plane Mn(III) sites^40^. It is possible that these sites are susceptible to reduction by carbohydrates, resulting in loss of catalytic reactivity with protein.

The ^1^H NMR analysis of the degradation of cellobiose and trehalose by birnessite revealed that several new resonances appeared over time. One such resonance corresponded to formate (^1^H chemical shift ~8.45ppm). Previous studies have shown that small organic acids, such as formate, are produced during the oxidation reaction of natural organic matter with birnessite^17^ and our observations show that the reaction of carbohydrates with birnessite also results in the production of formate (Table 1). Thus, formate produced by the reaction of natural organic matter with birnessite is likely partially derived from the degradation of carbohydrates present in natural organic matter. The degradation of carbohydrates or natural organic matter to smaller organic acids by birnessite highlights the potential role that mineral oxides could play in the conversion of large, modestly soluble organic compounds, to small highly soluble constituents of dissolved organic carbon and the transfer of such carbon through the soil matrix. In anoxic conditions, such as fens, formate is metabolized to other compounds, including acetate, propionate, and methane, making formate transport a potentially important process in carbon cycling^41^.

Our data show that the presence of carbohydrates could impact the abiotic degradation of other biomolecules that are present in the environment. This observation could be particularly relevant where proteins (exoenzymes) are secreted by microorganisms to degrade biological macromolecules and mobilize nutrients. The process of synthesizing and exporting these exoenzymes requires investment of significant energy resources^42^ and mineral surfaces that bind and inactivate exoenzymes are a potential energetic sink for these organisms. Extending the useful lifetime of these proteins in soil environments would be of value to these microorganisms^42^. The presence of carbohydrates, either produced by the microorganisms themselves, such as extracellular polymeric substances (EPS), or via the action of cellulases or related cellulosic biomass degrading enzymes, could increase the persistence of active enzymes in soil by protecting those enzymes from abiotic degradation catalyzed by reactive mineral surfaces. Alternatively, the active production of birnessite by microorganisms has been observed^43^ and may be another pathway by which microorganisms accelerate the mobilization of nutrients by oxidizing organic compounds to smaller, more soluble compounds. Fungi have been found to convert Mn(II) to reactive Mn(III), which subsequently oxidizes forest litter, aiding in the degradation of the litter^21^. Birnessite is formed during this process and our data suggests a subsequent role where birnessite could continue to further oxidize organic matter after it is partially broken down by reaction with Mn(III).

Soils are composed of a wide range of organic matter and mineral components. Our data show that these minerals can react differently with organic matter, such as carbohydrates, and result in different outcomes. In the case of the strongly oxidizing mineral oxide birnessite, which is common in soils of humid ecosystems, the mineral is capable of oxidatively degrading the carbohydrates. This observation is consistent with previous results that have shown that birnessite can oxidatively modify small organic compounds^18–20^. However, a redox inactive phyllosilicate, such as kaolinite, does not induce significant degradation of these carbohydrates under the conditions employed in our experiments.

Previously, we have demonstrated that birnessite can cleave the peptide bond in a model protein, likely via a hydrolytic mechanism, and that birnessite did not appear to participate in oxidation of the protein. Taken together, these results indicate that birnessite can act as both an oxidant in chemical reactions, as well as catalyze hydrolysis reactions. Consequently, the outcome of organic matter interactions with birnessite also depends on the composition of organic matter present. These data highlight the role that the mineral matrix could play in the complex soil environment. The types of minerals present and the types of organic components present can have a direct impact on the abiotic reactions that occur with these minerals, which in turn impact the degradation of organic constituents with ramifications for carbon turnover in soils. Of particular interest here is the disaccharide trehalose, as it is a common constituent of biofilms^27^. As a nonreducing sugar, trehalose is highly resistant to heat and extreme pH^44^. It has previously been posited that, in trehalose-producing organisms, this compound may serve as a buffer against stresses and as a protein stabilizer^45–47^. Some organisms accumulate trehalose to concentrations of 7% or more of their dry weight^25^, indicating that processes that require high concentrations of trehalose are biologically relevant. Our results show that high concentrations of trehalose and cellobiose can inhibit abiotic protein degradation by birnessite, suggesting that trehalose in biofilms and other extracellular organic substances produced by microorganisms may also be able to function as a protectant against abiotic oxidative degradation of exoenzymes. Such a mechanism would be particularly useful for saprotrophic fungi in forest ecosystems with moist soils enriched in pedogenic manganese oxides, and, given the importance of forest ecosystems for the functioning of the biosphere, certainly warrants further investigation.
